# Nasal Carriage of Methicillin Resistant *Staphylococci* in Healthy Population of East Sikkim

**DOI:** 10.4103/0970-0218.58403

**Published:** 2009-10

**Authors:** Devjyoti Majumdar, Ankur Barua, Barnali Paul

**Affiliations:** Department of Microbiology Sikkim Manipal Institute of Medical Sciences 5^th^ Mile, Tadong, Gangtok, Sikkim-737 102, India

## Introduction

Methicillin resistant *Staphylococcus aureus* (MRSA) strains were initially described in 1961 and emerged in the last decade as one of the most important nosocomial pathogens. MRSA is a strain of *S. aureus* that has developed resistance to methicillin and other beta *β-* lactamase-resistant penicillins and cephalosporins.(1) MRSA infections have recently been identified in the community, which raised a question of whether these infections were transmitted from hospital, or they were caused by different resistant strains. The sharp increase in the prevalence of MRSA acquired infections in many communities had led to the consideration of outpatients as a source of infection in an institution.(2) Epidemiology of MRSA in the community is little understood or not studied at length. A few reports on MRSA in the healthy population of Nigeria, USA, Canada, Pakistan and Japan are available in the world literature. Till the beginning of study no report on the prevalence of MRSA in the community in India was available. Case reports of community acquired MRSA infections had been increasing since last 3 years in the tertiary care level hospitals in Gangtok of East Sikkim. Hence, a study was undertaken to determine the prevalence of MRSA among healthy subjects in the community in Gangtok of East Sikkim in India.

## Materials and Methods

### Background information:

Gangtok with geographic-locations of (Lat/Lon Bounding Box: North=27.333332 South=27.333332 East=88.61667 West=88.61667 and altitude of 1547 m) has a population of 550,000. There are two tertiary care level hospitals, one government and the other private, at a distance of 5 miles apart in East Sikkim.

### Study design:

Community based cross-sectional survey.

### Study period:

Two months (March 2005 - April 2005).

### Sample size:

One Nasal swab from each of a total of 280 apparently healthy individuals was collected for the study.

### Selection method:

At the beginning, a spot-map of dwellings of different areas of East Sikkim was prepared. Then each distinguished village and nodal areas were identified in it and numbered serially. From these numbers, five spots (areas) in the map were randomly selected through lottery method. Thus, the areas selected for survey were - Deorali-Daragaon, Metro-point, Sarswati temple area, Loomse and Ranipool in East Sikkim. Households in the selected survey areas were numbered serially and specific number of households in each area (as calculated by power analysis from software package of epi-info version windows 2000) was chosen according to PPS method. Individual households were selected by using the random number table. Only one individual from each household was selected for the study through lottery method.

### Study participants:

Only one individual was included in the study from each household. Selection was done through lottery method after arranging all household members in ascending order of age. Since, *Staphylococcus aureus* (*S. aureus*) was frequently found as a part of the normal human micro flora, children below 13 years of age were excluded from the study. Persons who had been admitted in a hospital in the preceding 12 months or had used any antibiotic during that period or worked in a health care center were also not included in this study.

### Materials used for the survey:

Sterile cotton-swabs, Sterile test tubes, Nutrient agar (HiMedia Laboratories Private Ltd.), Mueller-Hinton agar (HiMedia Laboratories Private Ltd.) supplemented with 4% NaCl, Oxacillin disk-1μg (HiMedia Laboratories Private Ltd.), Control strain NCTC 6571 (ICMR, Dibrugarh), Other reagents for catalase, oxidase, Coagulase, phosphates, DNAse and sugar fermentation tests.

### Data collection procedure:

Nasal swabs were collected by sterile, dry cotton swabs from anterior nares of each nostril of a subject, inserting the swab and then gently rotating the swab three times.(3) The swabs were immediately placed in test tubes for further processing in the laboratory. All the isolates were tested for coagulase production following standard procedures. *Staphylococci spp* isolated were tested for Methicillin resistance by using modified Stokes same plate comparative disc diffusion method(2,3) using 1μg Oxacillin disk. Mueller-Hinton agar with 4% NaCl medium was used to detect Oxacillin resistance, incubated at 35°C for 24 hours.(4) Zone diameter of the test strain was measured in millimeter with a scale. Strains were classified as resistant or sensitive following standard procedure.

### Data analysis:

The collected data was tabulated in spreadsheets of Microsoft Excel version Office 2000 and analyzed by Epi Info version windows 2000.

## Results and Discussion

*S. aureus* nasal carriage rates in various populations have been investigated in the developed countries with temperate climate(5) but no such study among healthy population had been reported from India so far. Researchers reported that nasal carriage of *S. aureus* varied in different communities. The results of the present study showed that nasal carriage of staphylococci was as high as 88.2% and in 52.2% cases, S. aureus were isolated. Out of 129 *S. aureus* isolates, 31(24%) isolates were Oxacillin resistant and these are referred as MRSA. The prevalence of MRSA in the apparently healthy community of East Sikkim was estimated to be of 11.1%. A total of 129(46.1%) among 280 healthy individuals screened were nasal carriers of *S. aureus*. Similar findings were reported by M.S. Anwar *et al.* (2004) in their study in Lahore, Pakistan who screened 1024 and 636 apparently healthy persons from urban and rural area respectively for nasal carriage of *S. aureus* and MRSA reported that in urban areas prevalence of nasal carriers of *S. aureus* was estimated to be of 16.99%, but in rural areas it was 11.32%. In urban areas prevalence of nasal carriers of MRSA was found to be 22.98% as against 11.11% in rural areas.(2) In a study by A. Lamikanra *et al.* (1985) it was observed that 56.4% of healthy Nigerian group of students who were nasal carriers of *S. aureus*.(5) Out of 247 staphylococcal isolates, 118 (48%) were Coagulase Negative *Staphylococci* (nasal carriers of CNS) and among them, 26(22%) CNS was found to be resistant to oxacillin, referred to as Methicillin Resistant Coagulase Negative *Staphylococci* (MRCNS).

Sex-wise break up of nasal carriers of CNS and MRCNS did not show any significant difference [χ^2^=0.04, *P*=0.835 (Yates corrected)] in the rates of nasal carriage among male and female carriers. This finding was contrary to that observed in the study done in Nigerian population where females harbored *S. aureus* significantly more often than males.(5)

MRSA nasal carrier rate in the community was high (26.6%) in age group of (20-40) years of age and less (10%) in age groups below 20 years and above 40 years. It was also higher in the age group below 40 years of age (24.75%).

MRSA nasal carriage was lower in Ranipool (16.2%) and higher in Loomse (38.5%) than in other areas. But these differences are statistically not significant [χ^2^=1.72, *P*=0.156 (Fisher exact 2-tailed)]. An area-wise analysis of Methicillin sensitive vs. resistant strains of CNS show that statistically there was no significant difference in nasal carriage rate of MRCNS in areas near a tertiary care level hospital and away from the hospital. Since, acute and recurrent infections with *S. aureus* and MARSA are a possibility of developing drug resistant staphylococcal strains in the community, there is an urgent need to investigate the cause for a higher rate of MRSA nasal carriage in this study area. It is also recommended that epidemiological studies including genotyping are required to understand in detail, the dynamics of spread of MRSA and MRCNS in this community.
